# An Analysis of Knee Injuries in Rugby League: The Experience at the Newcastle Knights Professional Rugby League Team

**DOI:** 10.1186/s40798-019-0206-z

**Published:** 2019-07-24

**Authors:** George Elias Habib Awwad, Jennifer Helen Coleman, Christopher James Dunkley, David Craig Dewar

**Affiliations:** 1 John Hunter Hospital, Newcastle, NSW Australia; 2 Newcastle Knights Rugby League Club, Newcastle, NSW Australia

**Keywords:** Sports Medicine, Rugby, Sports injuries

## Abstract

**Background:**

Epidemiologic data in professional sport is becoming an increasingly valuable tool in identifying frequently occurring injuries and developing strategies to reduce their occurrence. Currently, there is a paucity of literature on the epidemiology of knee injuries in professional male rugby league players.

**Methods:**

We retrospectively reviewed medical records from a single male professional rugby league team (Newcastle Knights), competing in Australia, and evaluated knee injuries and time to return to play.

**Results:**

In total, 89 knee injuries occurred, with an injury incidence of 616.7 injuries per 1000 players. The most frequently occurring knee injuries were medial collateral ligament (416.7 injuries per 1000 players) and chondral/meniscal injuries (416.7 injuries per 1000 players). For all injury types, being tackled was the most common mechanism of injury, and the median time to return to play was 1 day. Anterior cruciate ligament injuries accounted for the longest time to return to play (median 236.0 days).

**Conclusion:**

Medial collateral ligament and chondral/meniscal injury types were the most frequent injuries; however, anterior cruciate ligament injuries accounted for the most time missed from sport despite being less common. Professional male rugby league players incur similar knee injury types compared to rugby union based upon our study and other similar studies.

**Electronic supplementary material:**

The online version of this article (10.1186/s40798-019-0206-z) contains supplementary material, which is available to authorized users.

## Key Points

- MCL and chondral/meniscal injuries account for most of the injuries seen in rugby league.
- Injury patterns and time to return to play are similar to players in rugby union.
- Larger prospective studies are needed to further examine these findings.

## Background

Epidemiologic data that pertains to a specific code of sport helps to identify frequently occurring injuries within that sport. This data can then be used to establish the aetiology and mechanisms of these injuries, develop and implement strategies to reduce their occurrence, and then re-assess the effectiveness of these changes [1]. Rugby league is a contact sport played internationally and popular predominantly in the Eastern states of Australia. There are 13 players on each team and the game is played on a rectangular field. In rugby union, there are 15 players per team, scrums are larger, and tackles are followed by the formation of rucks and mauls. Both rugby league and union are extremely gruelling contact sports, with alternating periods of high and low-intensity activity. However, there have been several rule changes to rugby league over the years that have made it a more free-flowing and taxing game. These have included the introduction of a maximum tackle count to prevent teams from holding the ball for long periods, replacing scrums with ball handovers reducing the number of stoppages during the game, and more recently the introduction of extra-time for drawn games, thereby extending their length.

Previous studies have examined the distribution of injury types in professional rugby league, and these have demonstrated that lower-limb and knee injuries are amongst the most common injury-type sustained [2, 3]. Despite the high rate of knee injuries seen in rugby league, there is a paucity of literature on the epidemiology of such injuries in professional male players, with current studies undertaking little analysis of knee injury subtypes in this population [2, 4]. However, other similar codes such as rugby union [5–7], Australian-rules football [8, 9], American football [10, 11], and soccer [12, 13] have a greater amount of data. For example, studies in rugby union have shown that knee injuries cause the most time missed from playing and training than injuries compared to other body parts [5].

The aim of our study was to evaluate the type and characteristics of knee injuries in a professional male rugby league team, mechanisms of injury, interventions required, and the time to recovery and to compare this to similar data in the literature.

## Materials and Methods

All players who played for the Newcastle Knights in the National Rugby League (NRL) competition in Australia between 2015 and 2017 were included in the study. Their medical records were reviewed, and all knee injuries occurring during their time at the club were analysed. Informed consent was obtained prior to accessing the medical records of participants of the study. Additional informed consent was obtained from all individual participants whose identifying information was included in this article. Ethics committee approval was also obtained from the local regulatory authority to perform the study.

Players were included in the study if they sustained a knee injury whilst playing or training in the NRL team/first-team squad. These injuries were classified by the team doctor and physiotherapists according to the Orchard Sports Injury Classification System [14] and recorded into an electronic database (Smartabase). The type of data recorded was based upon the *NRL injury definitions and data collection procedures* (Additional file 1; reproduced with permission from the NRL) and included player demographics, as well as injury data from games and training. These injuries were then grouped into broader categories of anterior cruciate ligament (ACL), medial collateral ligament (MCL), posterior cruciate ligament (PCL)/posterolateral corner (PLC), chondral/meniscal, patellofemoral joint (PFJ)/extensor mechanism, or other minor injuries.

Players were excluded from the study if they were not in the NRL team/first-team squad as training regimens and playing schedules were different for players in different squads. Players were also excluded if the injury sustained occurred whilst playing for another club as medical records of these injuries were not available for review. Finally, players sustaining an injury that did not directly involve the knee (e.g. hamstring injury) were also excluded.

The variables that were evaluated included the type of knee injury (ACL, MCL, PCL/PLC, chondral/meniscal, PFJ/extensor mechanism, or other minor injury), player demographics, details of injury (in game/training, contact, illegal play), primary mechanism of injury (getting tackled, tackling, running, changing direction, twisting, direct blow, insidious, other or unknown)**,** recurrence, whether operative treatment was required, and the time taken to return to play (RTP).

The definitions of injury and injury recurrence were as per guidelines supplied by the NRL, entitled *NRL injury definitions and data collection procedures* (Additional file 1). An injury was defined as any physical complaint, which was caused by the participation in rugby league training or a match, which required the need for medical attention or resulted in time loss from rugby league activities. Illegal play was defined as any injury caused by play resulting in a foul or penalty. RTP was defined as the time from onset of injury until the time the player was deemed fit enough for selection and not requiring any training modifications. The decision to return to play was made on a case-by-case basis by the medical team (physiotherapists and team doctor in conjunction with any consulted medical professionals).

All player data was collected, de-identified, and analysed in a Microsoft Excel spreadsheet. Data were reported as means and medians and rounded to a single decimal point. Injury incidence was also calculated and recorded as injuries per 1000 players.

## Results

In total, 60 players were included in the study that had played for the Newcastle Knights professional rugby league team in the NRL in seasons 2015 to 2017. All injuries sustained by players during their time at the club were evaluated. Players were on average 24.1 years of age with a body mass index (BMI) of 29.2. The majority of players in the study suffered a knee injury at an incidence of 616.7 injuries per 1000 players (Table 1).

**Table 1:** Demographic data for all injuries

Total number of knee injuries	Total number of players	Total number of players suffering knee injury	Proportion knee injuries in squad (%)	Incidence (injuries/1000 players)	Number of different types of knee injuries	Average BMI (range)	Average age (range)
89.0	60.0	37.0	61.7	616.7	21.0	29.2 (24.6–33.2)	24.1 (17.0–35.0)

*BMI* body mass index

### Injury Types

The most frequently occurring knee injuries were MCL and chondral/meniscal injuries. Other/minor injuries also occurred frequently; however, ACL injuries occurred the least frequently (Table 2). Of the three ACL injuries in our study, one was a graft rupture in a player who had previously sustained a primary ACL rupture at the club, and the other was a tibiofemoral dislocation in which the player also suffered a lateral collateral ligament tear and common peroneal nerve palsy. Players with PFJ/extensor mechanism injuries were the youngest and had the greatest BMI as compared with the other injury types.

**Table 2:** Demographic data of injury types

Type of injury	Total number of type	Proportion (%)	Incidence (injuries/1000 players)	Average BMI (range)	Average age (range)
MCL tear	25.0	28.1	416.7	29.4 (24.6–32.9)	23.6 (18.0–32.0)
ACL^#^	3.0	3.4	50	28.2 (27.8–29.0)	21.3 (19.0–24.0)
PCL/PLC	8.0	9.0	133.3	29.0 (26.3–32.0)	25.1 (18.0–33.0)
Chondral/meniscal	25.0	28.1	416.7	28.4 (26.0–32.9)	24.7 (19.0–29.0)
PFJ/extensor mechanism	11.0	12.4	183.3	30.1 (26.0–32.9)	20.9 (17.0–29.0)
Other/minor*	17.0	19.1	283.3	28.8 (26.0–33.2)	23.5 (19.0–35.0)

*BMI* body mass index, *MCL* medial collateral ligament, *ACL* anterior cruciate ligament, *PCL/PLC* posterior cruciate ligament/posterolateral corner, *PFJ* patellofemoral joint

*I njuries included: ITB friction syndrome, Baker’s cyst, joint capsule irritation, pes-anserine bursitis, Hoffa’s fat pad impingement, contusion, knee pain

^#^Injuries included: ACL graft rupture, tibiofemoral dislocation

### Mechanisms of Injury

More injuries occurred during a game than during training, with 2% due to illegal play. The majority of injuries were contact in nature, with two of the three ACL injuries being due to contact (Table 3).

**Table 3:** Injury details (game/training, illegal play, contact) as a function of injury type

Type of injury	Number injuries during game	Proportion (%)	Number injuries during game due to illegal play	Proportion (%)	Proportion (%)	Proportion (%)
MCL tear	19.0	76.0	0.0	0.0		88.0
ACL	3.0	100.0	0.0	0.0		66.7
PCL/PLC	6.0	75.0	0.0	0.0		62.5
Chondral/meniscal	12.0	48.0	2.0	16.7		48.0
PFJ/extensor mechanism	3.0	27.3	0.0	0.0		9.1
Other/minor	7.0	41.2	0.0	0.0		35.3
All injuries	49	55.1	2	2.2		53.9

*MCL* medial collateral ligament, *ACL* anterior cruciate ligament, *PCL/PLC* posterior cruciate ligament/posterolateral corner, *PFJ* patellofemoral joint

Primary injury mechanisms were divided into 10 categories as listed in Table 4. The most common mechanism was getting tackled (25.8%), closely followed by tackling (20.2%) and insidious causes (20.2%). The majority of both MCL tears and chondral/meniscal injuries were due to getting tackled, and the majority of PFJ/extensor mechanism injuries were due to insidious causes.

**Table 4:** Primary mechanism of injury as a function of injury type (%)

Type of injury	Getting tackled	Tackling	Running	Changing direction	Twisting	Direct blow	Insidious	Other (jumping, kneeling, kicking)	Unknown
MCL tear	44.0	32.0	0.0	4.0	4.0	4.0	4.0	0.0	8.0
ACL tear	33.3	33.3	0.0	0.0	0.0	0.0	0.0	33.3	0.0
PCL/PLC	25.0	37.5	12.5	12.5	0.0	0.0	12.5	0.0	0.0
Chondral/meniscal	28.0	16.0	0.0	12.0	4.0	4.0	12.0	8.0	16.0
PFJ/Extensor mechanism	9.1	9.1	9.1	0.0	0.0	0.0	72.7	0.0	0.0
Other/minor	5.9	5.9	29.4	5.9	0.0	17.6	29.4	5.9	0.0
All injuries	25.8	20.2	7.9	6.7	2.2	5.6	20.2	4.5	6.7

*MCL* medial collateral ligament, *ACL* anterior cruciate ligament, *PCL/PLC* posterior cruciate ligament/posterolateral corner, *PFJ* patellofemoral joint

### Recurrent Injuries

There were 73 new injuries sustained and 16 recurrent injuries. One player sustained an ACL graft rupture 2 years following the initial ACL reconstruction. PFJ/extensor mechanism injuries had the lowest recurrence of any injury, with only one of 11 recurring (Table 5).

**Table 5:** New and recurrent injuries by injury type

Type of injury	Number new	Proportion (%)	Number recurrent	Proportion (%)
MCL tear	21	84	4	16
ACL tear	2	66.7	1	33.3
PCL/PLC	7	87.5	1	12.5
Chondral/meniscal	20	80	5	20
PFJ/extensor mechanism	10	90.9	1	9.1
Other/minor	13	76.5	4	23.5
All injuries	73	82	16	18

*MCL* medial collateral ligament, *ACL* anterior cruciate ligament, *PCL/PLC* posterior cruciate ligament/posterolateral corner, *PFJ* patellofemoral joint

### Operative Treatment

Overall, the majority of players suffering a knee injury in our study did not require surgery, with only 12 undergoing operative treatment. All ACL tears underwent surgical treatment, with one player who sustained a tibiofemoral dislocation and subsequent multi-ligament knee injury requiring LCL reconstruction and delayed ACL reconstruction. All MCL tears, PCL/PLC tears, and other/minor injuries were managed non-operatively. One player with a PFJ/extensor mechanism injury required surgery after sustaining a patella dislocation and undergoing arthroscopy for removal of loose bodies (Table 6).

**Table 6:** Surgical treatment of injuries

Type of injury	Number surgery required	Proportion surgery required (%)
MCL tear	0.0	0.0
ACL	3.0	100.0
PCL/PLC	0.0	0.0
Chondral/meniscal	8.0	17.6
PFJ/extensor mechanism	1.0	9.1
Other/minor	0.0	0.0
All injuries	12	13.5

*MCL* medial collateral ligament, *ACL* anterior cruciate ligament, *PCL/PLC* posterior cruciate ligament/posterolateral corner, *PFJ* patellofemoral joint

### Return to Play

The average time taken to RTP from all injuries was just under 3 weeks, and the median value was 1 day. ACL injuries accounted for the longest time to RTP. At the time of publication, the player suffering a tibiofemoral dislocation was still undergoing rehabilitation and had not yet returned to play. The other player who sustained an ACL injury returned to playing first-grade rugby league after his initial rupture but was still in rehabilitation after his subsequent graft rupture. PCL/PLC injuries (median 31.0 days) had the second longest median RTP. Players suffering chondral/meniscal and PFJ/extensor mechanism injuries and other/minor injuries had the lowest median times to recovery (Table 7).

**Table 7:** Time to return to play of injuries (days)

Type of injury	Average time to RTP	Median time to RTP (range)
MCL tear	19.1	14.5 (0.0–104.0)
ACL	236.0	236.0 (236.0–236.0)*
PCL/PLC	37.6	31.0 (0.0–237.0)
Chondral/meniscal	5.4	4.0 (0.0–32.0)
PFJ/extensor mechanism	37.9	0.0 (0.0–83.0)
Other/minor	2.9	0.0 (0.0–60.0)
All injuries	20.7	1.0 (0.0–237.0)

*RTP* return to play, *MCL* medial collateral ligament, *ACL* anterior cruciate ligament, *PCL/PLC* posterior cruciate ligament/posterolateral corner, *PFJ* patellofemoral joint

*2 of the 3 ACL injuries in our study had not returned to play at the time of the study; hence, the range is of a single value

## Discussion

Knee injuries occurred in the majority of subjects in our study (37 of 60 players), with MCL tears and chondral/meniscal injuries accounting for most of these injuries. Just over half of all injuries occurred during games (55.1%), with being tackled the most common mechanism of injury. The majority of players suffering any knee injury were treated non-operatively (86.5%), with all ACL injuries undergoing surgery. ACL tears caused players to miss the most time from games (median 236.0 days).

After reviewing the literature, there were no other directly comparable studies focusing on the epidemiology of knee injuries in professional male rugby league. A study by Gibbs et al. [2] identified 141 injuries of all types occurring at an Australian professional rugby league club over a 3-year period and found that knee injuries were the most common injury sustained (24.1%). Whilst they did not specify each type of knee injury, they stated that MCL injuries were the most common (10.6%), ACL injuries occurred in 4.3%, and knee lateral capsule occurred in 2.1% of the players. These were the only subtypes of knee injury listed, and hence, the results are difficult to compare to our own study.

Whilst there were no directly comparable studies in rugby league, our results were comparable to a similar study in rugby union players by Dallalana et al. [5]. They evaluated 211 knee injuries in 12 clubs in the English Premiership (a professional male rugby union competition in England) over 2 seasons. Knee injuries occurred more often in match play than during training (an incidence of 11.0 vs 0.16 injuries per 1000 player-hours). They found that MCL (28.9%), other/small injuries (27.0%), and chondral/meniscal injuries (18.5%) were the most common injuries sustained. These proportions are similar to those seen in our study; however, chondral/meniscal injuries were more prevalent in our study compared with rugby union players (28.1% vs 18.5% respectively). The mechanisms of injury were similar to those seen in our study, although tackling was more common than getting tackled as a cause of injury in rugby union (the opposite to our study). Interestingly, the authors of the rugby union study defined an injury as those causing a player to miss training or game activity for a period of at least 24 h, which was slightly different to that in our study. It was unclear who decided on RTP in the rugby union study; however, the authors state that medical personnel reported all knee injury episodes, so presumably, these personnel also determined when a player was fit to RTP. In our study, the physiotherapists and team doctor in conjunction with any consulted medical professional made this decision. Bearing this information in mind, time to RTP was similar for most injuries in both studies.

In our study, MCL and chondral/meniscal injuries accounted for 56.2% of the knee injuries sustained and were the most frequent injury types. Whilst the median time to RTP for players with chondral/meniscal injuries was only 4.0 days, it took players more than 3 times as long to recover from MCL injuries (median 14.5 days, with an average time of 19.1 days). In rugby union, MCL injuries are also the most common injury seen [5], as well as in an American football [15], and form a high proportion of injuries in Australian-rules football [8, 16]. In rugby union and Australian-rules football, the average time missed from MCL injuries is 32 days and 25.2 days respectively. However, chondral/meniscal injuries account for a larger amount of average time missed in both rugby union and Australian-rules football (41 days and 43.4 days respectively vs. 5.4 days in our study), whilst this disparity may be explained by the different playing styles and player exposures in the codes, as well as the fact that our study analysed a smaller dataset from a single team unlike the Australian-rules football and rugby union studies.

Some studies have investigated the effectiveness of prophylactic bracing to prevent ligamentous knee injuries, particularly in college American football players, given the high rate of knee injuries seen in this sport [17]. However, the data showing benefit of such braces is not conclusive [18–21]. Furthermore, there has been concern that restricting knee motion may increase ipsilateral ankle injuries [22], as well as the potential for laceration and contusion type injuries to the player themselves and their opponent. Hence, the feasibility of players being able to wear such braces whilst playing rugby league remains questionable.

Australian-rules football has been more heavily studied, and the injury surveillance programmes are more developed, than those in rugby league. Studies of knee injuries in Australian-rules football have shown that knee cartilage injuries had the highest incidence followed by MCL and ACL injuries [8]. ACL injuries occurred more frequently in Australian-rules football studies than in our study, or than in other rugby league/union studies. In Australian-rules football, ACL injuries are usually non-contact in nature occurring during cutting or pivoting manoeuvers [23]. Non-contact ACL injuries are also more frequent in other sports such as basketball, soccer, and handball [24–26]. However, in both our own study and a similar study in rugby union [5], the majority of ACL injuries were due to contact. One of the ACL injuries in our study occurred whilst getting tackled, and the other occurred whilst performing a tackle. In the study by Dallalana et al., getting tackled caused most contact ACL injuries. This disparity in the mechanism for ACL injuries between rugby union, league, and Australia-rules football may reflect the fact that both rugby league and union are played on a smaller field with more direct and forceful collisions, as well as having a unique style of tackling, that make contact ACL injuries more likely. Given the high incidence of ACL injuries seen in Australian-rules football, there has been research studying the factors associated with ACL injuries in this football code. Non-contact ACL injuries have been shown to occur more frequently on fields with rye grass compared to other grass types [27]. Higher rates of ACL injuries have also been seen in those stadia located in climates with high evaporation and low rainfall prior to play [28]. This data may be applicable to rugby league and could be used in the prevention of such injuries with better field preparation and grass selection for playing surfaces.

In terms of mechanism of injury, both getting tackled and tackling accounted for nearly half (45.8%) of the knee injuries sustained. It is unclear whether the injuries themselves occurred at the point of contact, or after the initial impact during the twisting and landing phases of the tackle. The heavy-contact nature of rugby league is such that these mechanisms will most likely continue to account for a significant proportion of knee injuries sustained, unless drastic rule changes are made. However, there is scope for improved teaching of tackling techniques, and the ability of a player to safely “absorb” a tackle from an opponent, in an effort to reduce the number of knee injuries associated with these mechanisms.

Almost half of all injuries sustained in our study (44.9%) occurred during training. This included 72.7% of PFJ/extensor mechanism injuries, 52% of chondral/meniscal injuries, and 48.8% of other/minor injuries. These results differ from those reported by Dallalana et al. [5], where only 15.6% of rugby union players sustained injuries during training, and all injury types were more commonly sustained during games. Our results need to be compared with data from other rugby league clubs but certainly indicate that training practices need to be closely scrutinised to address this trend.

The strengths of our study include being limited to one club where all players were assessed in a similar manner by the same group of medical and allied health staff (including physiotherapists, a team doctor and consulting specialist orthopaedic surgeons). Our study is unique as it is the only such study in the literature investigating the epidemiology of knee injuries in professional male rugby league.

A limitation of our study was a low number of subjects and its retrospective nature. We also understand that whilst applicable to Australian rugby league, these results may differ internationally in countries with differing climates and conditions. We chose to limit our study to that of professional rugby league players, as it has been noted that injury rates and patterns differ based on whether rugby league is played at the amateur or professional level [29, 30]. We also limited the study to include players in the first team squad alone, as training and playing schedules were different at other squad levels within the club.

This study helps to increase the awareness of the most common and severe knee injuries in professional male rugby league players. This will enable better coaching and training practices, improve player well being, and could reduce the significant financial burden associated with these injuries by the use of targeted ‘pre-habilitation’ programmes. These programmes can utilise the information from this study to target the most common injury patterns seen and develop strength and conditioning exercises to reduce their occurrence. Pre-habilitation programs should also include exercises tailored to the individual player’s needs, accounting for their own injury history.

Overall, more epidemiological studies are needed with larger subject numbers to help identify whether the injury patterns and recovery times we have identified are indeed representatives of professional male rugby league. Analysing the data from other leagues around the world would also help determine whether the trends we have seen are unique to knee injuries seen in Australia, or whether they reflect injuries seen internationally as well.

## Conclusions

Knee injuries occur frequently in rugby league and affected more than half of the players in this study. Like in rugby union, MCL and chondral/meniscal injuries accounted for a high proportion of the knee injuries seen. ACL injuries were mainly contact in nature and accounted for the most time missed from play. Both being tackled and tackling were the most common mechanisms of injury, and a significant proportion of the injuries sustained occurred during training.

Longer-term and prospective studies are needed to gather more data and develop a clearer picture of the trends we have demonstrated. This will enable more accurate decision making for clubs in developing strategies to reduce the overall burden of these injuries, as well as the league in refining the rules of the game to protect players in the future.

## Additional file

Additional file1: NRL Injury definitions and data collection procedures. (PDF 453 kb)

## Data Availability

Please contact the author for data requests.

## Abbreviations

ACL: Anterior cruciate ligament
BMI: Body mass index
ITB: Iliotibial band
MCL: Medial collateral ligament
NRL: National Rugby League
PCL: Posterior cruciate ligament
PFJ: Patellofemoral joint
PLC: Posterolateral corner
RTP: Return to play
