# Supplementary material for: An Analysis of Knee Injuries in Rugby League: The Experience at the Newcastle Knights Professional Rugby League Team
Source: Sports Med Open. 2019 Jul 24;5:33. doi: 10.1186/s40798-019-0206-z (PMC6656824; doi:10.1186/s40798-019-0206-z)
Supplement: Supplementary file 1 [file 40798_2019_206_MOESM1_ESM.pdf]

1

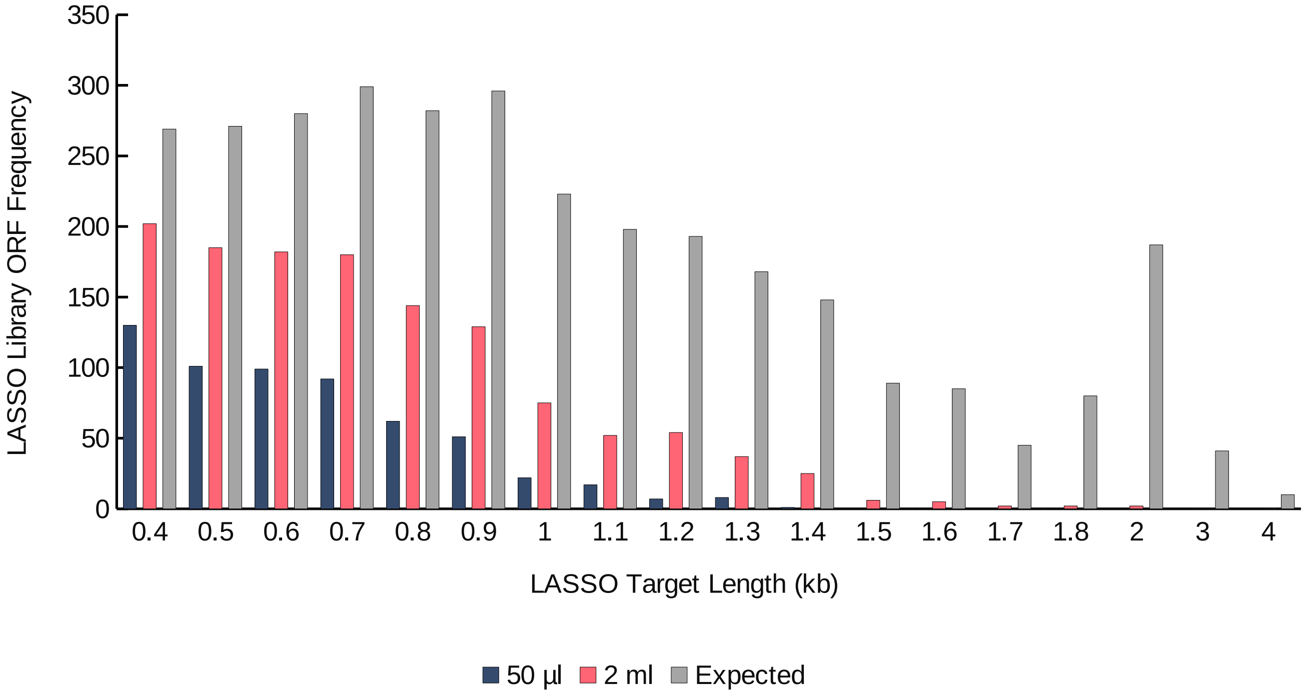

LASSO Library ORF Frequency
350
300
250
200
150
100
50
0
0.4
0.5
0.6
0.7
0.8
0.9
1
1.1
1.2
1.3
1.4
1.5
1.6
1.7
1.8
2
3
4
LASSO Target Length (kb)
50 µl
2 ml
Expected
